# Percutaneous Vertebroplasty in Adult Degenerative Scoliosis for Spine Support: Study for Pain Evaluation and Mobility Improvement

**DOI:** 10.1155/2013/626502

**Published:** 2013-10-24

**Authors:** Dimitrios K. Filippiadis, Panagiotis Papagelopoulos, Maria Kitsou, Nikolaos Oikonomopoulos, Elias Brountzos, Nikolaos Kelekis, Alexis Kelekis

**Affiliations:** ^1^2nd Radiology Department, University General Hospital “ATTIKON,” 1 Rimini Street, 12462 Athens, Greece; ^2^A Orthopedic Clinic, University General Hospital “ATTIKON,” 1 Rimini Street, 12462 Athens, Greece; ^3^B Anesthesiology Clinic, University General Hospital “ATTIKON,” 1 Rimini Street, 12462 Athens, Greece; ^4^Diagnostic and Interventional Radiology, 2nd Radiology Department, University General Hospital “ATTIKON,” 1 Rimini Street, 12462 Athens, Greece

## Abstract

We evaluate the efficacy-safety of percutaneous vertebroplasty (PV) as primary treatment in adult degenerative scoliosis. During the last 4 years, PV was performed in 18 adult patients (68 vertebral bodies) with back pain due to degenerative scoliotic spine. Under anaesthesia and fluoroscopy, direct access to most deformed vertebral bodies was obtained by 13G needles, and PMMA for vertebroplasty was injected. Scoliosis' inner arch was supported. Clinical evaluation included immediate and delayed studies of patient's general condition and neurological status. An NVS scale helped assessing pain relief, life quality, and mobility improvement. Comparing patients' scores prior to (mean value 8.06 ± 1.3 NVS units), the morning after (mean value 3.11 ± 1.2 NVS units), at 12 (mean value 1.67 ± 1.5 NVS units), and 24 months after vertebroplasty (mean value 1.67 ± 1.5 NVS units) treatment, patients presented a mean decrease of 6.39 ± 1.6 NVS units on terms of life quality improvement and pain relief (*P* = 0.000). Overall mobility improved in 18/18 (100%) patients. No complications were observed. During follow-up period (mean value 17.66 months), all patients underwent a mean of 1.3 sessions for facet joint and nerve root infiltrations. Percutaneous vertebroplasty in the inner arch seems to be an effective technique for supporting adult degenerative scoliotic spine.

## 1. Introduction

 Adult scoliosis is a term referring to spinal deformity with a Cobb's angle on frontal plain equal or greater to 10° which appears on adult patients whose skeletal maturity is complete [1]. Throughout the literature, several classification schemes exist for degenerative scoliosis (Simmons classification, Aebi system, Faldini working classification system, Schwab system, and the Scoliosis Research Society system) [2]. Clinical presentation of adult scoliosis includes pain, sagittal imbalance, claudication upon standing or walking, and neurological deficits including individual roots or the whole cauda equina with bladder and rectal sphincter dysfunction [3, 4]. Conservative treatments include pain medication with analgesics, nonsteroid anti-inflammatory drugs, and muscle relaxants, physical therapy, chiropractic care, bracing, and epidural/selective nerve root/facet joint infiltrations, but no doubt all these techniques are governed by moderate success [5]. Surgical treatment, on the other hand, has well established its efficacy and consists of posterior, anterior, or double anteroposterior approach, fusion, decompression, or osteotomy [6, 7]. However, although efficient, surgical approach due to its character and the increased age of the patients seems to be governed by notable mortality rate (34.3 per 1000 patients) and a 39% general complication rate [7, 8]. 

 Percutaneous vertebroplasty was introduced by Galibert and Deramond (1984), and the first report ever was published in 1987, concerning the treatment of a painful and aggressive vertebral body hemangioma in the cervical spine [9]. During the technique, a needle is inserted in the vertebral body of interest under percutaneous approach and imaging guidance. Once in position, through the trocar of the needle, polymethyl-methacrylate (PMMA) is injected solidifying the fractured vertebra and significantly reducing pain with consequent mobility improvement [10–17].

 Based on percutaneous vertebroplasty's efficacy in pain reduction, application of this technique to the most deformed vertebral bodies was evaluated in a consecutive series of patients suffering from back pain due to adult degenerative scoliosis.

## 2. Material and Methods

 All patients were informed about their inclusion in the study. Furthermore, they were informed upon the technique itself as well as possible benefits and complications, and they signed a written consent form to both the study and the procedure. The study received approval from the review board of our institution.

### 2.1. Patient Selection and Evaluation

 During the last 4 years, percutaneous vertebroplasty (PV) was performed in 68 vertebral bodies of 18 adult patients (age range 44–92 years) with back pain due to degenerative scoliotic spine. 

**Figure 1:**
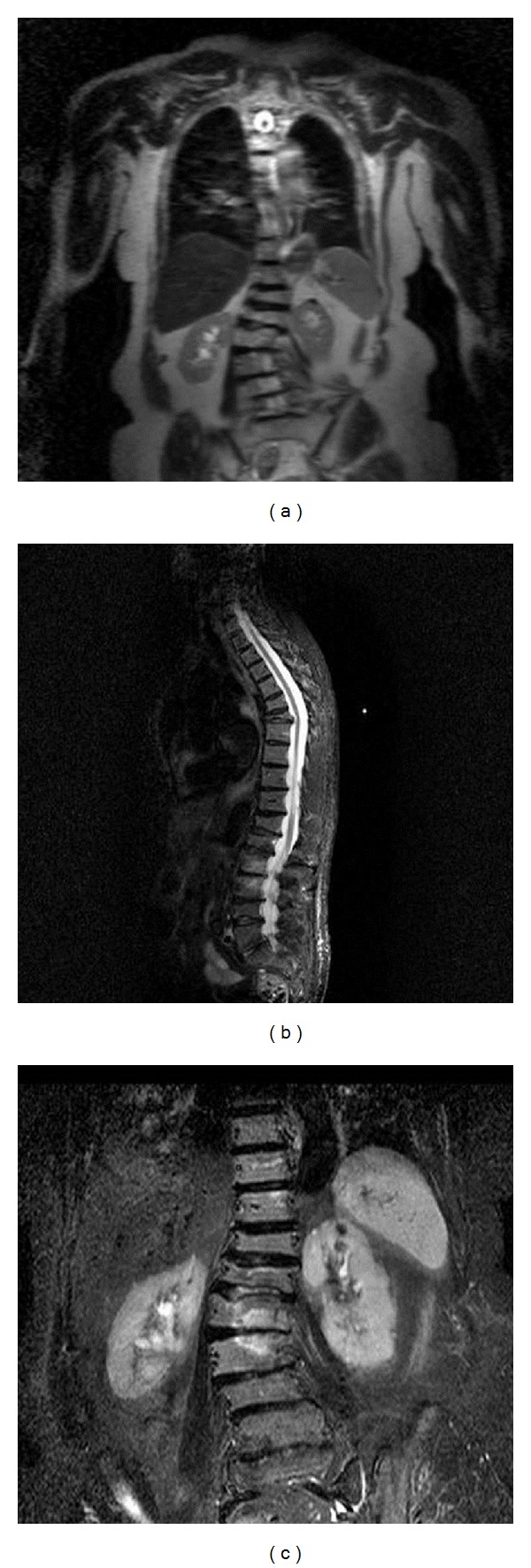
T2-weighted coronal sequence (a), sagital (b), and coronal (c) STIR sequence demonstrating scoliosis with the convex towards the right side and bone edema at L3, L3, and L1 vertebral bodies (in the inner side of the scoliotic arch). Notice the fracture line at L1 vertebral body under the upper end plate.

 Inclusion criteria included adult patients capable of providing consent capable of complying with the study protocol, and suffering from back pain due to adult degenerative scoliosis. Pain was unresponsive or minimally responsive to conventional treatments for at least 3 months. Clinical and imaging examination defined proper patient selection. Percutaneous vertebroplasty was performed on sites where local percussion over the posterior elements of the involved vertebral body elicited pain, and this positive spinous process percussion was combined with imaging modalities verification (presence of bone oedema at the level of interest on STIR sequences of magnetic resonance scan) (Figure 1).

 Each patient underwent evaluation of all imaging studies, physical/clinical examination, and coagulation laboratory tests at least 24 hours prior to PV. Preoperational imaging included X-rays (according the national care system in our country, this is the standard examination when a patient is admitted to a hospital for low back pain) and recent (up to 3 months old) multiplanar MRI (T1W, T2W, STIR). In selected cases where MRI was contraindicated (e.g., presence of cardiac pacemaker) bone scintigraphy combined with Computed Tomography scan was performed.

 Exclusion criteria for the procedure included lack of positive percussion of spinous process combined with presence of bone oedema at the level of interest, uncontrolled bleeding disorders, any ongoing systemic or spinal infection, myelopathy, and/or neurological deficit. Significantly reduced vertebral body height (vertebra plana), destruction of the posterior vertebral wall, and retropulsed bone were not considered exclusion criteria as long as there were not any neurological symptoms. 

**Figure 2:**
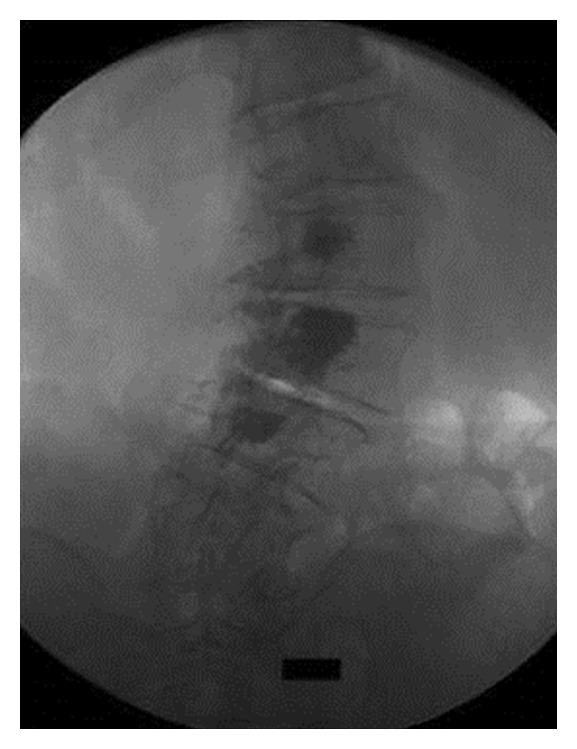
P-A fluoroscopic projection at the end of the vertebroplasty session in a different patient. Notice the PMMA distribution at the inner side of the scoliotic arch.

 Detailed description of the vertebroplasty technique (Figure 2) is beyond the scope of this paper. In any case, the guidelines described in CIRSE's and SIR's Standards of Practice document upon vertebroplasty were followed [18, 19].

**Figure 3:**
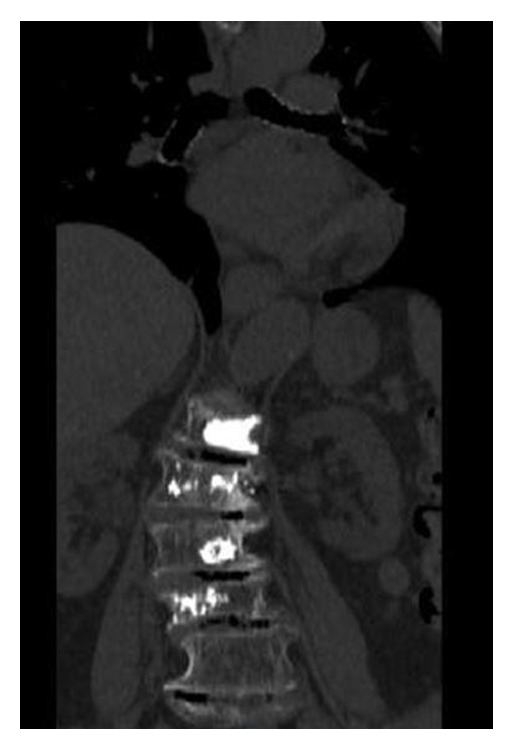
Computed Tomography scan, coronal reconstruction (different patient) after vertebroplasty. There are no cement leakages and PMMA is distributed in the inner side of the scoliotic arch. Notice the multilevel intradiscal vacuum phenomenon due to extensive spinal degeneration.

 CT scan assessed implant's position after treatment. All patients underwent control CT during the first 24 hours after PMMA injection (Figure 3). Clinical evaluation included immediate and delayed follow-up studies of patient's general condition and neurological status. All patients were evaluated before and after PV for pain, mobility, and overall satisfaction with the technique.

### 2.2. Outcome Measures

 Pain prior, the day after vertebroplasty, and during the 2-year follow-up period was recorded by means of NVS questionnaires. Numeric Visual Scale (NVS) is a 10 cm scale from 0 to 10 divided into ten equal parts (with a number corresponding to each part), upon which the patient subjectively assigns his/her pain on a score of minimum 0 (no pain) to maximum 10 (worst pain patient can imagine). In addition, the inventory contains questions concerning the pain itself and its influence upon patient's activity (sleep, occupation, housework, and walking) and mobility impairment. 

 We considered as statistically significant improvement in numeric pain scores a decrease of at least 4 NVS units. Pain-free patients were considered the ones with NVS score of 0.

### 2.3. Statistical Analysis

 All patients included in the study met the inclusion criteria.

Mean value, median value, and standard deviation of the self-reported pain scores prior, and after vertebroplasty were evaluated using related samples Wilcoxon signed-rank test and paired samples *t*-test. The threshold defining statistical significance was *P* < 0.05.

Statistical analysis was performed using SPSS for Windows (versions 17.0 and 21.0). IBM SPSS Sample Power 3 was used to calculate the statistical power of the study. 

## 3. Results

 The mean volume of injected cement for all 68 treated vertebral bodies was 2.624 ± 0.9328 cc (range 1.0–6.0 cc).  Table 1 demonstrates the distribution of treated vertebral bodies and a correlation of location to the amount of injected PMMA cement (Table 1). The vast majority of treated vertebral bodies (42/68 vertebral bodies) were found in the thoracolumbar junction (from T11 to L2 levels).

**Figure 4:**
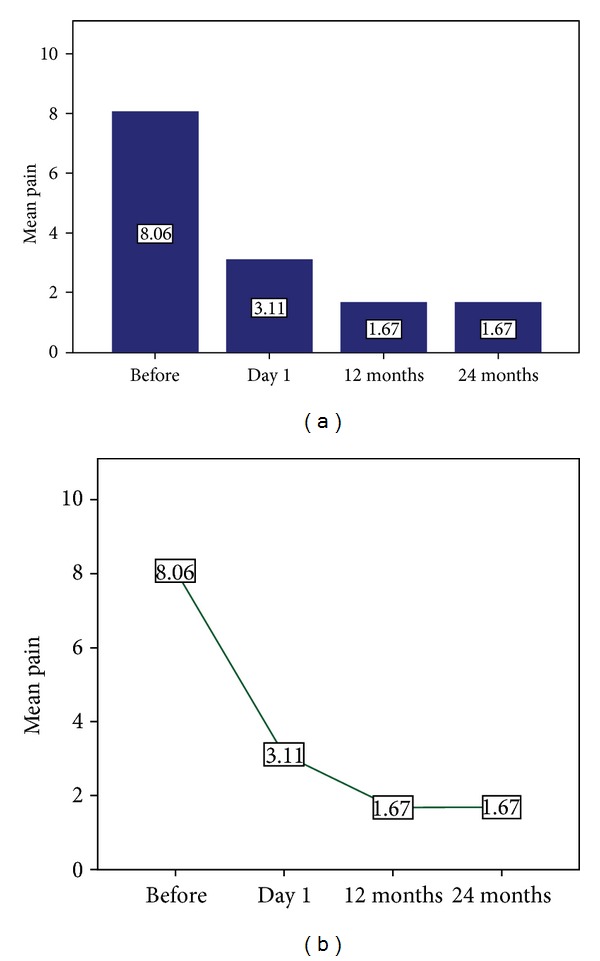
Two charts illustrating the progress of pain reduction throughout the follow-up period (prior, the morning after, at 12, and 24 months) in the patients included in our study.

 Comparing the patients' scores prior to vertebroplasty (mean value 8.06 ± 1.3 NVS units), the morning after (mean value 3.11 ± 1.2 NVS units), at 12 (mean value 1.67 ± 1.5 NVS units), and 24 months after vertebroplasty (mean value 1.67 ± 1.5 NVS units) treatment, patients of our study presented a mean decrease of 6.39 ± 1.6 NVS units on terms of life quality improvement and pain relief (*P* = 0.000) (Figure 4).

Overall mobility improved in 18/18 (100%) patients.

There were no clinically significant complications noted in our study.

**Figure 5:**
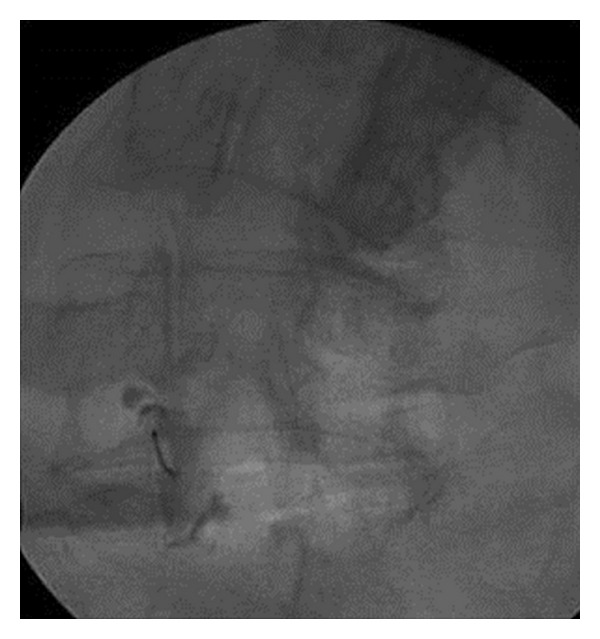
Scottie dog (45° degrees oblique) projection in a patient with symptomatic adult degenerative scoliosis (pain at L1, L2, and L3 level treated with percutaneous vertebroplasty). At 6 months followup, patient reported pain distributed at the level of L5-SI left facet joint which was treated with fluoroscopy-guided infiltration (injectate containing long acting corticosteroid and local anesthetic). Contrast medium injection under fluoroscopy verifies the intra-articular needle placement.

 During the follow-up period (mean value 17.66 months), all patients underwent a mean of 1.3 sessions for facet joint (Figure 5) and nerve root infiltrations. Character and distribution of pain were examined during the physical/clinical examination of the patient and were completely different from the original pain for which the patient was initially treated for.

According to IBM SPSS Sample Power 3, the statistical power of our study is 100%.

## 4. Discussion

 Adult scoliosis is an important and common causative agent of severe debilitating back pain affecting the quality of life and physical function of 1.4–12% of population worldwide and 6% of the population over the age of 50 years [20–22]. Primary (*de novo*) degenerative scoliosis involves spinal deformity that develops and becomes symptomatic after skeletal maturity of the patient is complete and finally secondary degenerative scoliosis due to miscellaneous causes including trauma and vertebral fractures, osteoporosis, and other metabolic bone diseases and iatrogenic causes [1, 23]. Pathophysiologic mechanism in a circular way involves the progressive segmental instability caused by asymmetric loading due to the asymmetric intervertebral disc and/or facet joints degeneration, resulting thus in asymmetric spinal deformity such as scoliosis and/or kyphosis which in turn induce further degeneration, asymmetric loading, and segmental instability [1]. This pathophysiologic circular mechanism results in osteophytes formation at the facet joints (spondylarthritis) and at the vertebral endplates (spondylosis), in hypertrophy and calcification of the ligamentum flavum which all contribute to spinal canal, central, and recessal spinal stenosis. Furthermore, the eccentric loading in the curvature apex or the facet joint asymmetric loading with arthritis or synovitis induces discogenic pain [1, 24–29]. 

 The most commonly reported symptoms include back pain, pain localised on the curve's apex or concavity and on the facet joints, sagittal imbalance, sciatica related to nerve root compression usually at the bottom of the curve, and claudication due to spinal central or recess stenosis [1, 22, 25]. Furthermore, neurologic deficit is usually related to herniated intervertebral disc fragment [1, 6]. 

 The decision for treating or not adult degenerative scoliosis and by what means is a complex one based upon many factors including patient's age, general health condition, and expectations as well as bone quality. Patients requiring treatment seem to constitute less than 1% of the screened population and less than 10% of those with curves greater than 10° [30, 31]. However, because patients over 50 years constitute the majority of this group, systemic diseases often coexist in addition to a possible significant surgical time with blood loss and other complications which result in longer and more intensive rehabilitation times [1, 32]. General complication rate is 39%, with 26% of patients undergoing a second operation [1, 7, 8]. Especially for posterior fusion and instrumentation for degenerative lumbar scoliosis, Cho et al. report complication rates of 68%, with abundant blood loss being a significant risk factor for early perioperative complications that included ileus, urinary tract infection, transient delirium, superficial infection, and neurologic deficit [33].

 The long-term outcomes, complications, and occasionally suboptimal results which accompany open surgery in adult degenerative scoliosis have led to the development of other treatment techniques that avoid an open surgery. Percutaneous vertebroplasty is a minimally invasive, image guided therapeutic technique which through direct usually transpedicular approach to the vertebral body uses acrylic cement (polymethylmethacrylate—PMMA) for pain reduction, mobility improvement, and stabilization of the vertebral column [34]. PMMA is a widely used material with well established effects as it has been used as a spinal stabilization adjunctive in open surgical treatment [35]. Even a small amount of PMMA (only 2 mL) can provide good pain relief which appears within 12–48 hours after the procedure [36]. Percutaneous vertebroplasty has gained wide acceptance worldwide because of its ability to significantly reduce pain resistant to conventional medical therapy in a rapid and durable fashion in the vast majority of treated patients. In addition, this minimally invasive image-guided procedure can be performed during a short hospital stay, with minimal patient discomfort [37, 38].

 MR scans with STIR (suppression of fat signal) sequences are very sensitive on illustrating bone oedema and played a pivotal role in patient inclusion in our study. As in the cases of arthritis and leukemia, pain due to bone edema can be attributed to a variety of factors including trabecular microfractures [39, 40]. Probably, this multicomponent of bone instability and/or insufficiency, intervertebral disc degeneration with progression to discogenic pain, and arthritis with inflammation of the articulation and synovitis is responsible for the produced pain in adult degenerative scoliosis. Thus, if pain is due mainly to the structural bone insufficiency of vertebral bodies, probably percutaneous vertebroplasty is a good solution for pain reduction and mobility improvement. On the other hand, when arthritis of the facet joints or discogenic pain is involved, epidural, selective nerve root or facet joint steroid infiltrations are probably the first interventional treatment to be used. 

Scoliotic spine looks like an arch and theoretically can be divided in three parts: outer, middle, and inner part. Any arch is principally balanced by two types of forces, compression forces and distraction forces. Geometrically, it is an established fact that for an arch to be mechanically stable and sustain weight the line of force between various parts of the arch (i.e., the site of balance between these various forces) must pass through the middle section. In the MRI of a scoliotic spine, bone edema lies in the middle and inner part of the curvature (imaging finding seen in the vast majority of the patients included in our study). Potential explanations for this include loading of the middle part (scoliosis' line of force) and trabecular microfractures (occurring either due to excess loading or due to microtraumas when subsequent vertebrae come close together). Since the maximum loading occurs in the middle and concave (inner) part of scoliosis, another potential explanation for bone edema might be that this is an expression of an elevated bone metabolism (remodelling).

 No doubt, as it is shown from our study, the pathophysiologic multicomponent of adult degenerative scoliosis requires combined therapies as well. Therefore, percutaneous vertebroplasty is performed for the pain due to bone insufficiency/edema/microfractures and is combined with percutaneous infiltrations for facet joint syndrome or neuralgia. In any case neurologic deficit is an emergency requiring surgical treatment, and vertebroplasty with or without infiltrations should be reserved for the rest of the cases. Clinical examination plays a pivotal role for proper patient selection. Patients with pain exacerbation during percussion of the spinous process should be treated with vertebroplasty. Those with pain exacerbated upon pressure at the level of the facet joint (facet joint syndrome) or upon the level of interspinous ligament (Baastrup disease) or with sciatica or other neuralgias should be directed towards other treatments.

 Up to our knowledge, this is the first study upon vertebroplasty in adult scoliosis. Limitations of our study include the small number of patients enrolled. Reasons for this include slow recruitment and patients' choice of other treatment regime. Another limitation is the lack of randomized control group for the assessment of vertebroplasty as a therapeutic regime for lumbar scoliosis and the fact that our study is not a prospective one. No doubt, extended studies with larger patient sample are necessary to support and prove these preliminary results.

## 5. Conclusion

Percutaneous vertebroplasty with PMMA seems to be an effective therapeutic technique for supporting adult degenerative scoliotic spine (especially in the inner arch). It can be proposed as an initial treatment in the scoliosis of the elderly prior to surgery and in combination with conservative treatment methods. Preliminary results report significant pain reduction and mobility improvement but further studies are required.

## Figures and Tables

**Table 1:** Anatomical distribution of treated vertebral bodies and correlation to the amount of injected PMMA cement (mL).

Location of treated vertebral bodies	Number of treated vertebrae/level	PMMA cement mean value (mL)	Standard deviation (SD)
Th9	1	1.5	
Th10	2	2.5	
Th11	6	1.967	0.5125
Th12	9	2.467	0.6928
L1	11	2.582	0.8818
L2	16	2.606	0.7496
L3	12	2.858	1.3222
L4	9	2.856	1.0525
L5	2	3.900	0.5657
